# Accidental acquisition of a rescued Japanese encephalitis virus with unspliced introns in the viral genome when using an intron-based stabilization approach

**DOI:** 10.1007/s00705-022-05697-z

**Published:** 2023-01-11

**Authors:** Ying Huang, Hongshan Xu, Shan Liu, Jiansheng Lu, Lili Jia, Yuhua Li, Rong Wang, Peng Yang, Yongxin Yu, Zhixin Yang

**Affiliations:** 1 Laboratory of Protein Engineering, Beijing Institute of Biotechnology, Beijing, 100071 People’s Republic of China; 2 Department of Arboviral Vaccine, National Institute for Food and Drug Control (NIFDC), Beijing, 102629 People’s Republic of China; 3 Department of Pathology, General Hospital of the PLA Rocket Force, Beijing, 100088 People’s Republic of China

## Abstract

The intron-based stabilization approach is a very useful strategy for construction of stable flavivirus infectious clones. SA_14_-14-2 is a highly attenuated Japanese encephalitis (JE) live vaccine strain that has been widely used in China since 1989. To develop safe and effective recombinant vaccines with SA_14_-14-2 as a backbone vector, we constructed the DNA-based infectious clone pCMW-JEV of SA_14_-14-2 using the intron-based stabilization approach and acquired the rescued virus rDJEV, which retained the biological properties of the parental virus. Unexpectedly, a rescued virus strain with altered virulence, designated rHV-DJEV, was accidentally acquired in one of the transfection experiments. rHV-DJEV showed up to 10^5^-fold increased neurovirulence compared with the SA_14_-14-2 parental strain. Genome sequencing showed that the inserted introns were still present in the genome of rHV-DJEV. Therefore, we think that the intron-based stabilization approach should be used with caution in vaccine development and direct iDNA immunization.

## Introduction

Flaviviruses are a group of single-stranded, positive-sense RNA viruses that cause worldwide epidemics with thousands of deaths annually, bringing great human and veterinary health burdens [1]. Construction of flavivirus infectious clones enables direct genetic manipulation of viral genomes, and this is a powerful tool that can be used in many aspects of virus research. However, some flavivirus infectious clones are very difficult to maintain in *E. coli* due to their predisposition to spontaneous rearrangements and/or frequent mutations. Various approaches, such as circular polymerase extension reaction, Gibson assembly, and intron-based stabilization, have been employed to overcome the instability problems of flavivirus infectious clones and to construct full-length cDNA clones successfully [2].

Japanese encephalitis is a disease of the central nervous system in humans caused by Japanese encephalitis virus (JEV), a member of the family *Flaviviridae*. The disease remains a pressing public health problem in the Asia-Pacific region [3]. The live attenuated JE vaccine SA_14_-14-2 was developed by the research group of Prof Yu Yongxin at NIFDC and licensed in 1989 in China. After the introduction of the SA_14_-14-2 vaccine into the national immunization program, a decline in JE morbidity became evident [4]. The vaccine was shown to have long-term immunogenicity, ideal protective efficacy, and only minor side effects after two doses. In recent years, research on novel flavivirus vaccines has targeted the production of chimeric vaccines using attenuated viruses or live vaccines as backbone vectors. [5–7]. With this background, we have investigated the use of SA_14_-14-2 as a vaccine backbone vector to deliver the structural proteins (prM and E) of other flaviviruses and obtain chimeric live-attenuated vaccine candidates.

In this study, we constructed a DNA-based infectious clone of the SA_14_-14-2 vaccine strain using an intron-based stabilization approach [8]. Upon direct transfection of cells with the infectious clone pCMW-JEV, a rescued virus, rDJEV, which retained the biological properties of the parental virus, was produced. However, we also found a rescued virus strain (designated rHV-DJEV) with altered virulence in one of the repeated transfection experiments. The neurovirulence test showed that rHV-DJEV had more than 10^5^-fold higher virulence than SA_14_-14-2. Sequencing the full-length genome of rHV-DJEV showed that the inserted introns were still present in the genome. The extra introns in the genome of the rescued virus might therefore be responsible for the increased neurovirulence of rHV-DJEV in mice. For the sake of safety, we recommended that the intron-based stabilization method to develop engineered viruses and prepare vaccines for direct infectious DNA (iDNA) immunization be used with caution.

## Materials and methods

### Cells, viruses, and animals

Vero cells were grown in minimal essential medium (MEM) supplemented with antibiotics and 10% fetal calf serum (FCS). BHK-21 cells were maintained in Dulbecco’s modified Eagle’s medium (DMEM) supplemented with 2-10% FCS. Cells were maintained at 37 °C in a humidified atmosphere containing 5% CO_2_. Vero and BHK-21 cells, the vaccine strain SA14-14-2, and the high-virulence JEV strain P3 were obtained from NIFDC. JEV is classified as a BSL2 agent according to China’s regulations on the laboratory biosafety management of pathogenic microorganisms. All experiments involving JEV were conducted in an approved, restricted-entry biosafety level 2 (BSL-2) laboratory.

Specific-pathogen-free weanling Kunming mice and guinea pigs were obtained from Beijing Laboratory Animal Center (Beijing, China). All animal experiments were carried out in accordance with the guidelines of the Laboratory Animal Care and Use Committee of the Beijing Institute of Biotechnology.

### Design and assembly of a DNA-based infectious clone of SA_14_-14-2

**Fig. 1:**
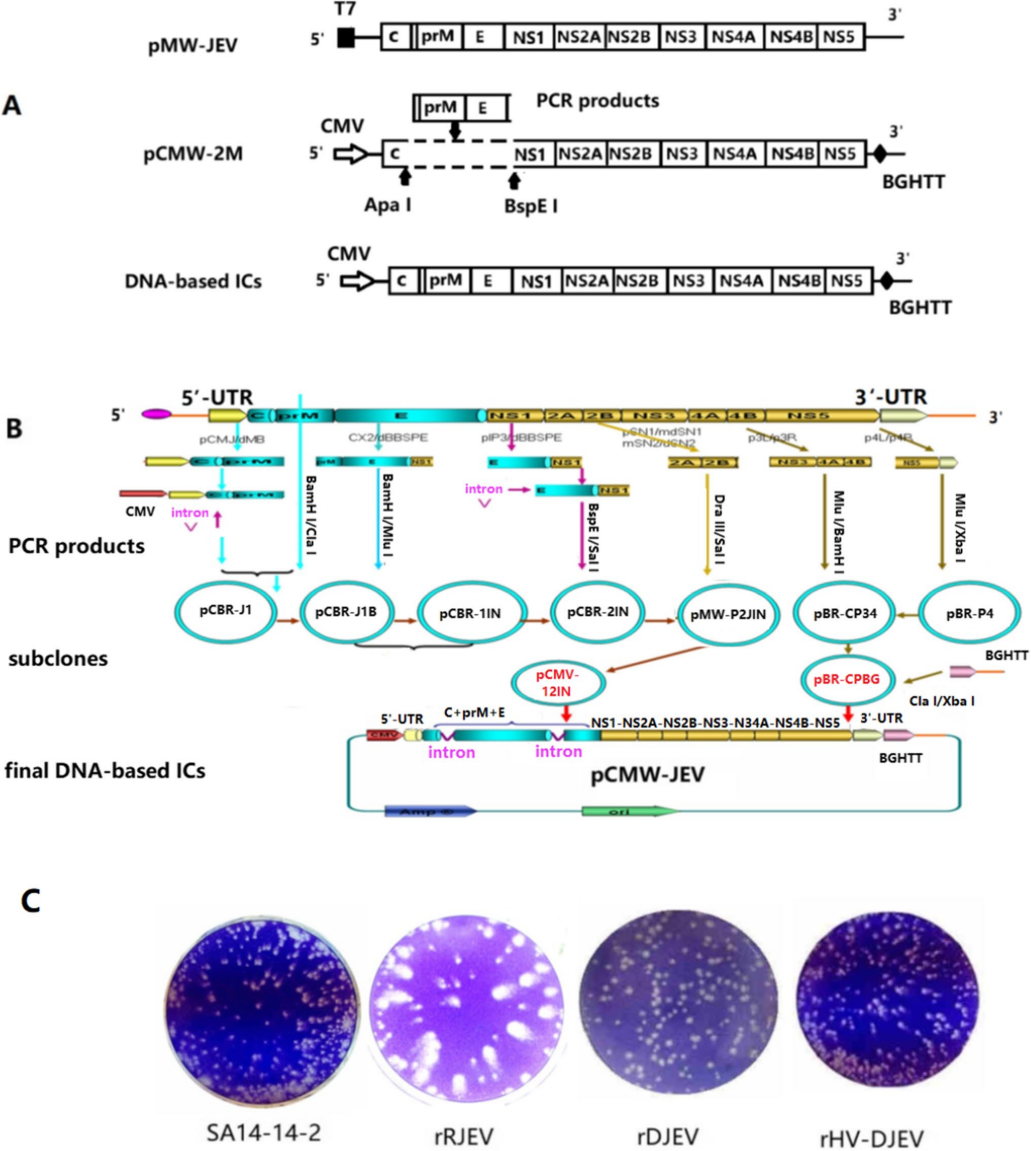
Construction and characterization of a DNA-based infectious clone of SA_14_-14-2. **A** Schematic diagrams showing the DNA-based infectious clone of SA_14_-14-2 designed on the basis of the JEV RNA-based clone pMW-JEV and the DNA-based replicon pCMW-2M. A dotted open box in pCMW-2M represents an in-frame deletion from nt 477 to nt 2414. The DNA-based infectious cDNA clone could not be produced in one step due to the instability of the plasmids. **B** An intron-based stabilization approach was used to construct a DNA-based IC designated pCMW-JEV. Two artificial introns were inserted into the SA14-14-2 genome at positions 354 and 2217. The nucleotide positions are based on the genome sequence of SA_14_-14-2 (GenBank accession no. D90195). **C** Plaque morphology of the SA_14_-14-2 parental virus and rescued viruses on BHK-21 cells. rRJEV, virus recovered from RNA-based clone pMW-JEV; rDJEV, P8 virus recovered from DNA-based clone pCMW-JEV; rHV-DJEV, highly virulent virus at P8 initially rescued from pCMW-JEV

DNA-based infectious clones (ICs) of JEV were designed based on JEV RNA-based infectious clone pMW-JEV and the DNA-based replicon pCMW-2M described previously [9]. The JEV prM-E genes were amplified from pMW-JEV by PCR and inserted into the Apa I and BspE I cleavage sites of pCMW-2M to construct DNA-based ICs. However, initial attempts to assemble the full-length cDNA clone in one step failed due to the instability of the plasmids in *E. coli* (Fig 1A).

To avoid the instability problems that frequently occur in the construction of flavivirus cDNA clones, we employed the intron-based stabilization approach, which was reported by Yamshchikov et al. [8]. As shown in Fig 1B, seven PCR-amplified cDNA fragments obtained using pMW-JEV as the template were sequentially inserted into the low-copy plasmid pMW-118 (Nikkon, Japan) to construct a SA_14_-14-2 DNA-based full-length infectious clone (designated pCMW-JEV). During the cloning steps, artificial introns were inserted into the JEV genome at nt 354 and at nt 2217, and silent mutations were introduced at nt 9392 and at nt 9403 of the JEV genome, creating a Kpn I restriction endonuclease site for use as a genetic marker. A human cytomegalovirus (CMV) promoter was fused to the beginning of SA_14_-14-2 genome, and a bovine growth hormone transcription termination (BGHTT) signal sequence was inserted immediately downstream of the last nucleotide of the genome to mediate termination of JEV transcripts at the natural 3′ end. All resultant subclones and the final pCMW-JEV IC were sequenced to monitor and correct nucleotide mutations.

### Infectivity of iDNAs in Vero cells

Transfection of Vero cells with pCMW-JEV was carried out using Lipofectamine 2000 Transfection Reagent (Invitrogen, Carlsbad, CA, USA) according to the manufacturer’s protocol. The cells in a 6-well plate were transfected with 2 µg of DNA per well. After 3-4 days of incubation, the supernatants were collected and clarified by centrifugation at 1000 × *g* for 20 min to obtain passage 1 (P1) progeny virus. Working stocks of P2 viruses were prepared by infecting Vero cells with the P1 virus at an MOI of 0.1 and harvested between 4 and 5 days postinfection when the cytopathic effect (CPE) was moderate. The resulting P2 viruses were harvested and amplified once to produce P3 viruses, and so on. As more additional passages were performed, the viruses were harvested and stored at -80 °C. Titers of the rescued viruses at different passages were determined on BHK-21 cells using a plaque assay.

### Virus neutralization (VN) assay

To examine the biological properties of the cDNA-derived viruses, a virus neutralization assay was performed as follows: Virus stock was prepared in tenfold serial dilutions, ranging from undiluted to 10^-5^, using sterilized PBS. The virus dilutions were mixed with an equal volume of a national standard JEV antiserum at known concentrations, with an unrelated national standard WNV antiserum as a negative control. The virus-serum mixtures were incubated at 37 °C for 90 minutes and then added to triplicate wells of 6-well plates containing confluent monolayers of BHK-21 cells with a volume of 200 µL per well. After 2 h of adsorption with gentle shaking every 15 min, the cells were overlaid with 5 ml of overlay medium containing methylcellulose. Five days later, plaques were visualized by staining with 0.5% crystal violet and counted. The neutralizing index (NI) was calculated as NI = (Tn-Tp+3)^10^, where Tn and Tp represent the remaining virus titers of 10^-1^ dilutions of viruses mixed with positive serum and 10^-4^ dilutions of viruses mixed with negative serum, respectively. A virus for which the NI value exceeded 1000 was considered to be specifically neutralized by the antiserum.

### Immunological identification tests

The supernatant from a P8 virus-infected cell culture was diluted 1000-fold with maintenance medium, and 100 µl of this dilution was inoculated onto confluent BHK-21 cell monolayers cultured in 6-well plates for 12 or 48 h. For immunohistochemical (IHC) staining, the cells were fixed in 100% methanol for 10 min at room temperature and then allowed to react sequentially with anti-JEV E mouse monoclonal antibody 2G2 (Uncover, Beijing, China), anti-mouse IgG (Zhongshan, Beijing, China), peroxidase-antiperoxidase (PAP) complex, and 3, 3'-diaminobenzidine (DAB) solution.

For immunofluorescence analysis (IFA), the cells were fixed with 4% paraformaldehyde and permeabilized with 0.2% Triton X-100. After blocking with 2% bovine serum albumin (BSA), the cells were incubated with mAb 2G2 as the primary antibody and a fluorescein isothiocyanate (FITC)-conjugated anti-mouse IgG as the secondary antibody (Santa Cruz, CA, USA). Cells were stained with Evans blue. The images were viewed and recorded using a confocal microscope under blue light at 450–480 nm.

A Western blot assay was used to detect the expression of the E protein. P8-virus-infected cells were cultivated for 48 h in serum-free medium. After 3-4 repeated freeze-thaw cycles, supernatants were collected and clarified by centrifugation at 1000 × *g* for 20 min, after which the supernatants were concentrated tenfold using a centrifugal filter units (Millipore, Billerica, MA, USA). Samples were separated by SDS-PAGE under reducing and non-reducing conditions, transferred to polyvinylidene difluoride membranes, and incubated with mAb 2G2, followed by HRP-conjugated anti-mouse IgG antibody (Zhongshan, Beijing, China). The bound peroxidase was visualized using an Immobilon Western blot detection system (Invitrogen, Carlsbad, CA, USA).

### Growth curves of the rescued viruses

The growth characteristics of clone-derived viruses were examined on Vero and BHK-21 cells. Subconfluent cells were infected at an MOI of 0.01 with the eighth passage of rescued viruses or SA_14_-14-2 as a control. Samples were collected at approximately 24-h intervals after infection. Viral progeny were harvested and frozen until all samples were collected. Virus yields at different time points were determined using plaque titration in BHK-21 cells.

### Detection of viremia in mice and guinea pigs

Groups of four 10–12 g mice were inoculated subcutaneously with 10^3^ plaque-forming units (PFU) of rHV-DJEV, rDJEV, SA_14_-14-2 vaccine strain, or virulent JEV P3 strain as a control. Two 250–300 g Hartley guinea pigs were inoculated intraperitoneally with 2.5 × 10^4^ PFU of each of these four viruses. Blood samples for the determination of viremia were collected daily from the orbital sinus of the mice or the heart of the guinea pigs for 7 days after infection. Undiluted or diluted (1:10, 1:100) pooled sera of each group were titrated by plaque assays on BHK-21 cells. Assays were performed in triplicate per dilution.

### Comparison of the virulence of the parental and rescued viruses

The neuroinvasiveness of the rescued viruses was assessed in Kunming weanling mice. Groups of four or six 10-2 g mice were inoculated by the subcutaneous (s.c.) route with tenfold dilutions of rescued viral stocks at passage 8 or parental SA_14_-14-2 virus. The inocula were back-titrated immediately after the injection procedure. One group of mice was injected with PBS only as a negative control. The LD_50_ values for neuroinvasiveness were calculated using the Reed and Muench method after 3 weeks of observation for the development of encephalitis and mortality.

The neurovirulence of the P1–P10 viruses was assessed in four or six 12-14 g Kunming weanling mice per group, which were inoculated intracerebrally (i.c.) with 30 µl of the virus that had been diluted tenfold in PBS. The virus inocula were immediately re-titrated. The mice were observed for 2 weeks for signs of encephalitis and death, and the LD_50_ values were then calculated.

### Genome sequencing

To determine the full genome sequence of rHV-DJEV, rHV-DJEV(P8) samples were obtained from cell culture supernatants and the brains of mice inoculated i.c. with P8 virus. Total RNA was extracted from each brain tissue or cell supernatant sample using TRIzol Reagent (Invitrogen, Carlsbad, CA, USA). The whole genome sequence of rHV-DJEV(P8) was determined, including the 5′ and 3′ termini, which were sequenced using a SMARTer RACE cDNA Amplification Kit (Clontech, Mountain View, CA, USA). To investigate whether nucleotide mutations had occurred during serial passage of rHV-DJEV in Vero cells, viral RNA was extracted at different passages and sequenced. The genomic sequence of rHV-DJEV was compared with the pCMW-JEV plasmid DNA sequence and the published nucleotide sequence of SA_14_-14-2 (GenBank accession no. D90195).

## Results

### Recovery of clone-derived viruses in Vero cells

To explore SA_14_-14-2 as a novel vaccine vector and facilitate the selection of viable chimeric viruses as vaccine candidates for other flaviviruses, the JEV DNA-based infectious clone pCMW-JEV was constructed. Because the instability of infectious clone constructs in *E. coli* made it hard to obtain full-length cDNA clones (Fig. 1A), two artificial introns were inserted into the JEV genome at nt 354 and nt 2217, using an intron-based stabilization approach (Fig. 1B).

As has been reported elsewhere [10, 11], introns introduced into flavivirus cDNA clones can be removed *in vivo* by splicing after transfection of susceptible cell lines, resulting in a rescued virus whose genome is identical to that of the original virus. The rescued virus rDJEV retained the biological properties of the parental virus in almost all of the transfection experiments (data not shown). However, we also found that one of the rescued viruses, rHV-DJEV, had altered virulence properties. Rescued rHV-DJEV virions were cultured and harvested on Vero cells. On day 3 posttransfection, a CPE was observed, which developed progressively from days 4 to 6. After additional passages in Vero cells, the titers of P2-P12 rHV-DJEV were in the range of 4.37-7.34 log PFU /ml. The plaques displayed a needle-tip-like shape, similar to that of the parental SA_14_-14-2. As shown in Fig. 1C, there was no difference in plaque size and morphology between the rescued viruses rDJEV and rHV-DJEV and the parental virus. Plaques produced by virus recovered from the RNA-based clone pMW-JEV exhibited size difference, indicating that the synthetic JEV RNA from *in vitro* transcription was not homogeneous and produced heterogenous populations of rescued virus.

### Immunological identification of rHV-DJEV

Standard virus neutralization assays are frequently used to identify viruses. In this study, A JEV antiserum was used to identify the cDNA-derived viruses, with the WNV antiserum as a control. As shown in Table 1, rDJEV and rHV-DJEV reacted using JEV antiserum, with NI titers exceeding 1000, whereas very little reactivity was observed with the WNV antiserum. The results confirmed rHV-DJEV to be JEV. However, the NI titers of the vaccine strain SA_14_-14-2 and rDJEV with JEV antiserum exceed 200,000, which is significantly higher than that of rHV-DJEV, indicating that rHV-DJEV differs from SA_14_-14-2.

**Table 1:** Serological identification of the clone-derived virus

Virus	Neutralization index with	
	National standard of WNV antiserum	National standard of JEV antiserum
SA_14_-14-2	85	≥ 251,189
rDJEV	57	≥ 225,893
rHV-JEV	33	6457

**Fig. 2:**
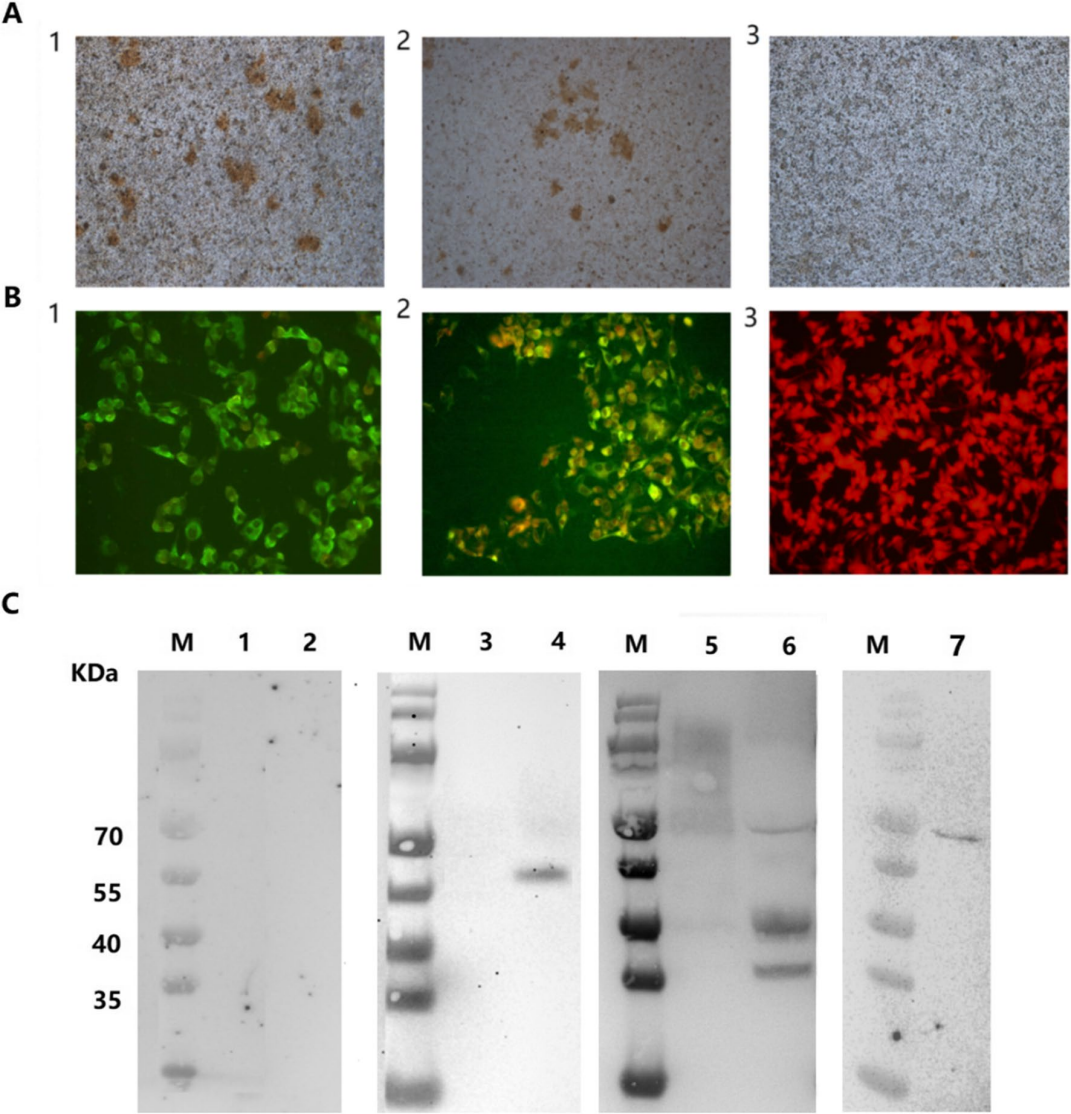
Biological characterization of the cDNA-clone-derived virus rHV-DJEV. **A** Immunohistochemical staining of BHK-21 cells infected with SA_14_-14-2 (1), rHV-DJEV (2), or mock infected (3) at 12 h postinfection. **B** At 48 h postinfection, SA_14_-14-2 (1), rHV-DJEV (2), and mock-infected (3) BHK-21 cells were tested by IFA for JEV E. **C** Western blots showing the recognition of the JEV E protein of viruses in the supernatants of cells infected with SA_14_-14-2 (lanes 3 and 4), rHV-DJEV at P8 (lanes 5 and 6), or rDJEV at P8 (lane 7), or mock infected (lanes 1 and 2). Lanes 1, 3, and 5: samples were loaded under reducing conditions. Lanes 2, 4, 6, and 7: samples were loaded under non-reducing conditions; Lane M, pre-stained protein molecular weight marker (Thermo Fisher Scientific, Waltham, MA, USA).

Virus at the eighth passage was used to infect BHK-21 cells, and at 12 h postinfection, positive signals were detected in rHV-DJEV-infected cells by IHC assay (Fig. 2A). The viruses readily infected the cells and formed multiple-cell foci. As shown in Fig. 2B, an IFA revealed that cells infected with JEV virions exhibited obvious fluorescence at 48 h postinfection, and there was no significant difference between the intensity of the positive signals obtained for rHV-DJEV and SA_14_-14-2.

A Western blot assay with electrophoresis performed under reducing and non-reducing conditions was used to analyze the JEV E protein expression from SA_14_-14-2 and rHV-DJEV. When using a reduced virion sample for SDS-PAGE, the antibodies did not recognize the E protein. Under non-reducing conditions, samples from supernatants of SA_14_-14-2- or rDJEV-infected cells were found to produce a 55-kDa band presumed to be the E protein, while samples from supernatants of rHV-DJEV-infected cells showed several clear positive bands with molecular masses ranging from 35 to 70 kDa. These results suggested that the envelope protein of rHV-DJEV differed from that of the original virus SA_14_-14-2.

### Growth properties of rHV-DJEV and SA_14_-14-2

**Fig. 3:**
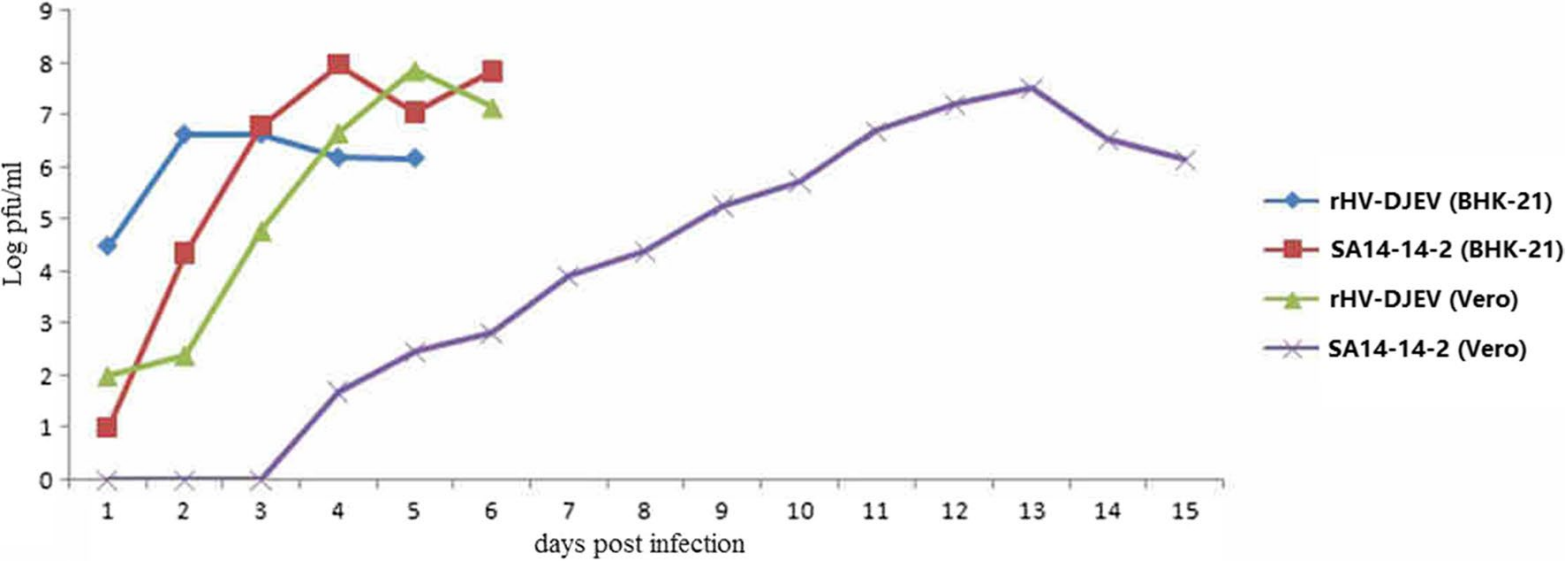
Virus growth kinetics of rHV-DJEV and SA_14_-14-2 on BHK-21 and Vero cells. Both cell lines were infected at a multiplicity of infection (MOI) of 0.01 with the eighth passage of the rescued virus rHV-DJEV or SA_14_-14-2 as a control.

To compare the growth characteristics of rHV-DJEV to those of the parental virus SA_14_-14-2, growth experiments were carried out in BHK-21 and Vero cells at a low multiplicity of infection (MOI). In both BHK-21 and Vero cells (Fig. 3), significant differences were observed between the viruses in terms of the maximal yield and duration of virus production. In BHK-21 cells, the virus yield of rHV-DJEV reached a peak at day 2 postinfection, while the yield of SA_14_-14-2 reached a peak titer at day 4. However, the maximal virus yield of SA_14_-14-2 (7.95 logPFU/ml) was 20-fold higher than that of the rescued virus. In Vero cells, rHV-DJEV also showed different growth kinetics when compared with the parental virus. rHV-DJEV accumulated rapidly and reached a peak at day 5. However, there was a significant delay in the replication of SA_14_-14-2 viruses, which continued for two weeks in Vero cells without a strong CPE. The titers of SA_14_-14-2 increased slowly and reached their peak at day 13.

### Viremia in mice and guinea pigs

To assess the peripheral replication of rHV-DJEV, levels and durations of viremia in the sera of mice and guinea pigs infected with wild-type and clone-derived viruses were measured. Table 2 shows the pattern of viremia in mice after inoculation with different viruses. In SA_14_-14-2-infected and rDJEV-infected mice, transient viremia was observed for only one day. In contrast, rHV-DJEV caused low viremia on day 2, which then increased, peaking at day 4, and clearance was observed 1-2 days later. In comparison, the virulent JEV strain P3 produced strong viremia. Virus titers in the serum reached 40 PFU/ml after infection and peaked around day 5 (3700 PFU/ml). All mice in the P3-injected group developed neurological symptoms at days 3–4 and died at day 6 or 7, consistent with the increased levels of viremia.

**Table 2:** Viremia of wild-type and clone-derived viruses in mice

Virus^a^	Virus titer in serum (PFU/ml)^b^						
	Day 1	Day 2	Day 3	Day 4	Day 5	Day 6	Day 7
JEV P3	40	105	150	330	3700	ND	ND
SA_14_-14-2	0	7	0	0	0	0	0
rDJEV	0	9	0	0	0	0	0
rHV-DJEV	0	18	28	30	0	0	0

^a^Mice at 10–12 g were inoculated subcutaneously with 10^3^ plaque-forming units (pfu) of rHV-DJEV, rDJEV, the SA_14_-14-2 vaccine strain, or the highly virulent JEV P3 strain.

^b^Pooled sera of each group were collected and titered by plaque assays on BHK-21 cells.

*ND* not determined

The levels and duration of viremia were also determined in guinea pigs. No viruses were detected in the sera of guinea pigs injected with SA_14_-14-2 or rHV-DJEV during the observation period of 10 days. In contrast, guinea pigs injected with the virulent P3 strain developed slight viremia, with virus titers of 10^6^ PFU/ml on day 1 postinfection. Viremia lasted for 2–3 days, with a peak titer of 1000 PFU/ml. The amount of virus in sera decreased dramatically at 4 days postinfection until no virus was detected. Guinea pigs in the P3 group also survived infection with no significant symptoms.

### Neurovirulence and neuroinvasiveness in mice

The neuroinvasiveness of rHV-DJEV(P8) was tested by subcutaneous (s.c.) inoculation of Kunming weanling mice and compared with that of SA_14_-14-2. The results are summarized in Table 3. All of the mice survived s.c. inoculation with SA_14_-14-2 and rHV-DJEV, even at the highest titers. This showed that rHV-DJEV is non-neuroinvasive in weanling mice, like its parental virus SA_14_-14-2.

**Table 3:** Neuroinvasiveness and neurovirulence of rescued viruses in weanling Kunming mice

Virus	Neurovirulence (i.c.)^a^			Neuroinvasiveness (s.c.) ^b^		
	Dose (pfu)	D/T	LD_50_	Dose (pfu)	D/T	LD_50_
rHV-DJEV	9.49 × 10^3^	4/4	16.8	1.04 × 10^5^	0/6	> 1.04 × 10^5^
	9.49 × 10^2^	4/4		1.04 × 10^4^	0/6	
	94.9	3/4		1.04 × 10^3^	0/6	
	9.49	2/4		1.04 × 10^2^	0/6	
	0.949	0/4		10.4	0/6	
SA_14_-14-2	1.1 × 10^5^	0/6	> 1.1 × 10^5^	3.65 × 10^5^	0/6	> 3.65 × 10^5^
	1.1 × 10^4^	0/6		3.65 × 10^4^	0/6	
	1.1 × 10^3^	0/6		3.65 × 10^3^	0/6	

D/T: deaths total

^a^Actual dose delivered intracerebrally (i.c.) in 0.03 ml of viral suspension to 12–14 g weanling Kunming mice

^b^Actual dose delivered subcutaneously (s.c). in 0.1 ml of viral suspension to 10–12 g weanling Kunming mice

Neurovirulence tests were performed in 12–14 g Kunming mice, which were inoculated i.c. with tenfold dilutions of viral stocks and observed for 2 weeks. As shown in Table 3, rHV-DJEV caused 100% mortality at a dose of 9.49 × 10^2^ PFU after i.c. inoculation and showed a high degree of neurovirulence, with an LD_50_ of 16.8 PFU. These results were not consistent with our expectations. The neurovirulence of the rescued virus rHV-DJEV was found to be to 10^5^-fold higher than that of SA_14_-14-2.

To assess whether the neurovirulence of rHV-DJEV had changed during growth in Vero cells, groups of four to six 12-14 g mice were inoculated i.c. with serial dilutions of P1–P10 viruses, and the LD_50_ value of rHV-DJEV at the different passages was calculated. Like rHV-DJEV at P8, viruses at the other passages exhibited a relatively high degree of neurovirulence, with the LD_50_ in the range of 16.5 to 59.9 PFU.

### Nucleotide sequence analysis of the rescued viruses

The SA_14_-14-2 genome contains 10,977 nucleotides. To identify nucleotide sequence differences between the rescued virus and the parental virus, the genome of rHV-DJEV was sequenced using nine overlapping RT-PCR products. The genomic termini were also determined using the rapid amplification of cDNA end (RACE) method. Analysis of the genome sequence of rHV-DJEV(P8) revealed three major differences in comparison to the consensus sequence of SA_14_-14-2: (i) The two introns that had been inserted into plasmid pCMW-JEV were unexpectedly still present in the viral genome of rHV-DJEV, (ii) a genetic marker with two silent mutations was present in the viral genome at positions 9392–9403 of rHV-DJEV, and (iii) two spontaneous nucleotide substitutions resulted in amino acid substitutions: one located in prM (prM82:Thr-Ala) and the other in NS4A (NS4A190: Ala-Asp).

The mutations at prM82 and NS4A190 were not found in the genome of rHV-DJEV virus at P1, and the sequence at both sites was identical to that of the parental virus SA_14_-14-2, indicating that the mutations had occurred during passage of the virus in Vero cells. The neurovirulence of the P1 virus was tested and confirmed to be also high, indicating that these two mutations had little influence on the virulence of rHV-DJEV.

## Discussion

Previously, we reported the construction of an RNA-based IC of SA_14_-14-2 named pMW-JEV [9]. JEV RNAs were transcribed *in vitro* from pMW-JEV and introduced by transfection into BHK-21 cells, and the virus rRJEV was rescued, as expected. However, the RNA-based IC had the limitation that a heterogeneous RNA population was always produced during the *in vitro* transcription procedure, and plaques of different sizes were observed in plaque assays (Fig. 1C).

To address this problem and simplify the procedure, the RNA-based clone pMW-JEV was converted to a DNA-based format. The construction of a DNA-based IC designated pCMW-JEV was accomplished using an intron-based stabilization approach, with two artificial introns inserted into the SA_14_-14-2 genome at positions 354 and 2217.

Upon transfection of Vero cells with cDNA, the added introns were spliced from the precursor nuclear messenger RNA (pre-mRNA) in the nucleus, resulting in a true copy of the viral RNA, which was transported to the cytoplasm. Like other rescued viruses that have been reported elsewhere [10–14], our rescued virus rDJEV retained the biological properties of the parental virus. However, we also accidentally found a rescued virus, termed rHV-DJEV, that was found to have altered virulence in the neurovirulence test.

Neutralization assays using JEV antiserum showed that rHV-DJEV reacted with the JEV antiserum with a neutralization index (NI) of 6457. IHC and IFA results showed that rHV-DJEV but could not be distinguished from SA_14_-14-2 in its ability to react with the anti-JEV E mAb 2G2. Western blotting further showed that the JEV E antigens of rHV-DJEV and SA_14_-14-2 differed in size and suggested that rHV-DJEV have a mixture of E proteins with different molecular weights.

Viremia in susceptible animal models is a key predictor of the severity of the virus disease [15]. In general, transient viremia is observed in the SA_14_-14-2-infected mice for only 1 day. In this study, a slight and transient viremia was observed in rHV-DJEV-infected mice, after which the virus was rapidly cleared, similar to the parental virus. No virus was detected in the sera of guinea pigs injected with SA_14_-14-2 or rHV-DJEV during the observation period of 10 days.

The neuroinvasiveness and neurovirulence of the rescued viruses was assessed in weanling Kunming mice. Like SA_14_-14-2, rHV-DJEV was found to be non-neuroinvasive in weanling mice. A surprising finding was the 10^5^-fold increased neurovirulence of rHV-DJEV relative to the parental virus SA_14_-14-2. As shown in Table 3, SA_14_-14-2 is avirulent, with a high LD_50_, as reported previously [4], while rHV-DJEV caused 100% mortality at a dose of 9.49 × 10^2^ PFU after i.c. inoculation and showed a high degree of neurovirulence, with an LD_50_ of only16.8 PFU.

Sequence analysis of the genome of the rescued virus at P8 identified a few differences, including two mutations related to Vero cell adaption that were not present in the plasmid pCMW-JEV. Earlier, Pletnev et al. reported the construction of an IC of tick-borne encephalitis virus (TBEV) [16] and showed that mutations occurred during amplification of the rescued virus in Vero cells. The spontaneous mutations might increase the replication efficiency of the virus and allow it to become more adaptive to the culture medium. However, such mutations can sometimes cause phenotype changes, including changes in growth characteristics and virulence [17]. Liu et al. demonstrated the genetic instability of an infectious-cDNA-clone-derived dengue type 4 virus during serial passage in Vero cells, with the neurovirulence of the virus increasing significantly following passage in Vero cells [18].

Subsequent tests verified that the two Vero-cell-adaptation mutations had little influence on the neurovirulence of rHV-DJEV, because the rescued virus at P1, without mutations at prM82 and NS4A190, also had high neurovirulence, with an LD_50_ of 42.4 PFU. Additional experiments were done to assess the virulence of the resulting rescued virus rDJEV. Similar to SA_14_-14-2, rDJEV showed no virulence in weanling mice after i.c. inoculation, even at high titers (LD_50_ > 6.25 × 10^6^ PFU). The complete genome of rDJEV was sequenced and compared with that of rHV-DJEV (P1), and the only difference was that rDJEV had no introns. These results suggest that the introns inserted into the genome of rHV-DJEV were responsible for the increased neurovirulence of rHV-DJEV in mice.

In an earlier study by González et al. [19], the researchers extracted cytoplasmic and nuclear RNAs and amplified the region covering the intron insertion site by RT-PCR after transfection with a full-length IC of the coronavirus transmissible gastroenteritis virus containing an intron. Bands of two different sizes were obtained, indicating that the efficiency of intron removal in viral RNAs transcribed *in vivo* from an intron-containing full-length cDNA was not 100%. We therefore speculated that a small number of RNA transcripts were left unspliced during nuclear transcription of pCMW-JEV. Although the translated product was not identical to the correct viral protein, this did not appear to affect the assembly of the virion, and an infectious virus was obtained. During subsequent virus cultivation and amplification, the virus entered the cells and released the nucleocapsid into the cytoplasm. Virion RNA containing the introns entered the next replicative cycle, producing progeny viruses that still contained introns in the genome.

Because the biological properties of rescued virions with unspliced introns are difficult to predict, the intron-based stabilization approach needs to be viewed with caution, especially in the development of engineered viruses for vaccine development and direct iDNA immunization.

## Data Availability

The data that support the findings of this study are available from the corresponding author upon reasonable request.
